# Establishment and Application of Duplex Recombinase-Aided Amplification Combined with Lateral Flow Dipsticks for Rapid and Simultaneous Visual Detection of *Klebsiella pneumoniae* and *Staphylococcus aureus* in Milk

**DOI:** 10.3390/foods14040573

**Published:** 2025-02-09

**Authors:** Ni Zhang, Laiwang Hou, Darong Li, Weiqing Lan, Yong Zhao, Xiaohong Sun

**Affiliations:** 1 College of Food Science and Technology, Shanghai Ocean University, Shanghai 201306, China; zhangni202103@163.com (N.Z.); lwhou_0922@163.com (L.H.); cysusan24@163.com (D.L.); wqlan@shou.edu.cn (W.L.); yzhao@shou.edu.cn (Y.Z.); 2 Shanghai Engineering Research Center of Aquatic-Product Processing & Preservation, Shanghai 201306, China; 3 Laboratory of Quality & Safety Risk Assessment for Aquatic Products on Storage and Preservation (Shanghai), Ministry of Agriculture, Shanghai 201306, China

**Keywords:** *Staphylococcus aureus*, *Klebsiella pneumoniae*, recombinase-aided amplification, lateral flow dipstick, rapid detection

## Abstract

*Staphylococcus aureus* and *Klebsiella pneumoniae* are significant and prevalent pathogens associated with bovine mastitis on dairy farms worldwide, resulting in severe infections in both dairy cows and, subsequently, human beings. Fast and dependable pathogen diagnostics are essential to minimize the effects of cow mastitis and human infections. The aim of this research was to develop a duplex recombinase-aided amplification (RAA) combined with the lateral flow dipstick (LFD) method, which was used for rapid, simultaneous detection of *S. aureus* and *K. pneumoniae*. The SKII culture medium for *S. aureus* and *K. pneumoniae* cocultivation was developed in this study. By optimizing the duplex RAA–LFD reaction conditions in terms of primer concentration, amplification temperature, and reaction time, the duplex RAA–LFD assay could successfully detect *S. aureus* and *K. pneumoniae* when the reaction was conducted at 39 °C for 20 min. The duplex RAA–LFD method demonstrated good specificity, exhibiting no cross-reactivity with other pathogens. In addition, the detection limit of the duplex RAA–LFD for *S. aureus* and *K. pneumoniae* was 60 fg of genomic DNA and 1.78 × 10^3^ and 2.46 × 10^3^ CFU/mL of bacteria in pure culture. Moreover, the duplex RAA–LFD technique is capable of identifying *S. aureus* and *K. pneumoniae* in artificially spiked milk samples even at very low initial concentrations of 1.78 × 10^1^ and 2.46 × 10^0^ CFU/mL, respectively, after 6 h of enrichment. The result of the actual samples showed that the total concordance rate of the duplex RAA–LFD method with the biochemical identification method and PCR method could reach 92.98~98.25% with high consistency. The results of this study indicated that the duplex RAA–LFD assay, which is a precise, sensitive, and simple field testing technique, can be used to identify *S. aureus* and *K. pneumoniae* and is expected to be used for disease diagnosis.

## 1. Introduction

*Staphylococcus aureus* and *Klebsiella pneumoniae* are two foodborne pathogens. These two pathogens have the ability to colonize and infect many different species and are one of the main reasons for mastitis in dairy cattle, which is often transmitted through water, feed, soil, farmers, and veterinarians who are in close proximity to the animals that have been colonized. This situation leads to substantial economic losses for dairy farms and poses serious health risks to humans [1,2]. Mastitis caused by *S. aureus* infection affects milk quality and adversely affects the reproductive efficiency of cows. Furthermore, *S. aureus* has been identified as one of the causative pathogens associated with food poisoning around the world [3]. Eating contaminated food with them may result in severe acute gastroenteritis, sepsis, or meningitis. *S. aureus* has been implicated in numerous cases of food poisoning associated with the consumption of contaminated raw milk and cheese [4,5,6]. *S. aureus* is responsible for over 240,000 cases of foodborne illness each year in the USA [7]. Bovine mastitis affected by *K. pneumoniae* leads to a significant reduction in milk production, resulting in an estimated annual loss of nearly USD 1 billion [8,9]. *K. pneumoniae* also causes a range of diseases in humans, including pneumonia, urinary tract infections, and bacteremia [10]. Dairy farmers may become colonized or infected with *K. pneumoniae* while managing dairy cattle. The mortality rate among immunocompromised individuals infected with *K. pneumoniae* can reach as high as 50% [11]. Thus, it is crucial to accurately and efficiently identify *S. aureus* and *K. pneumoniae* to minimize the impact of infections on both cows and humans.

The conventional culture method for detecting foodborne pathogens is time-consuming and labor-intensive. Moreover, it is difficult to differentiate bacteria with similar phenotypes using the conventional culture method. Polymerase Chain Reaction (PCR) demonstrates greater sensitivity in comparison to the traditional culture method. However, the requirement for thermal-cycling equipment and trained personnel limits its applicability in field inspection situations. Loop-mediated isothermal amplification (LAMP), developed in the latest times, has been developed to realize rapid and accurate pathogen detection. However, the LAMP assay requires four to six specific primers, which is complicated [12]. Recombinase-aided amplification (RAA) is highly sensitive and specific, which can complete nucleic acid amplification within 20 min under 37–42 °C [13]. This method has been developed for the detection of foodborne pathogens, as it does not necessitate complex instrumentation to maintain temperature [14]. This characteristic renders it more convenient in resource-limited settings compared to traditional molecular biology techniques.

**Figure 1:**
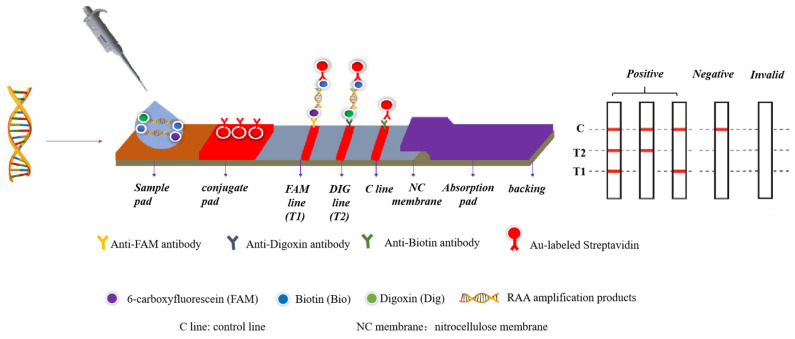
The working principle of the duplex lateral flow dipstick assay.

RAA produces target fragments under isothermal conditions by mimicking DNA amplification in vivo. At the beginning of the amplification reaction, recombinase binds primers with the participation of ATP to form complexes that can scan target DNA complementary to the primer sequences. Once localized to the homologous sequences, a strand-exchange reaction occurs and initiates DNA synthesis wherein the single-stranded DNA binding protein (SSB) binds to the displaced single strand to stabilize it [15]. Meanwhile, while the recombinase leaves, the DNA polymerase binds to the 3′ end of the primer and a new complementary strand is formed by undergoing strand elongation to realize the nucleic acid’s exponential amplification [16]. A variety of measures can be utilized to analyze the amplification products, such as agarose gel electrophoresis (AGE), fluorescent dyes, and lateral flow dipsticks (LFDs). A LFD is easy to operate and less dependent on the equipment [17,18]. The specific principle is as follows: firstly, chemical modifications (6-carboxyfluorescein (FAM) and digoxin (DIG)) were labeled at the 5′ end of the forward primer for two pathogens, respectively, and biotin was labeled at the 5′ end of the reverse primer for the two pathogens. The FAM duplex DNA–biotin and DIG duplex DNA–biotin of DNAs were obtained by RAA. The products flow forward with capillary action when added dropwise to the sample pad of the test strip. When passing through the binding pad on the LFD, they bind to the streptavidin-modified spherical Au Nanoparticles to form FAM duplex DNA–biotin–anti-biotin–AuNP and DIG duplex DNA–biotin–anti-biotin–AuNP complexes. Anti-FAM antibodies on the T1 test line trap the FAM duplex DNA–biotin–anti-biotin–AuNP complex, and anti-DIG antibodies on the T2 test line trap the DIG duplex DNA–biotin–anti-biotin–AuNP complex. The excess colloidal gold particles are trapped on the C line (control line) [19,20]. After 5–10 min of incubation, positive reactions exhibit a red color in the lateral flow dipstick for both the test and control lines. Conversely, negative reactions will display a red color solely on the control line (Figure 1). The RAA–LFD test has been employed for identifying many pathogens, including *Anguillid herpesvirus* [21], *Vibrio parahaemolyticus* [22], and *Phytophthora infestans* [23]. However, previous research has primarily concentrated on a single target. The duplex RAA method for simultaneous detection of *S. aureus* and *K. pneumoniae* has not been established.

In our research, a duplex RAA–LFD analysis approach was established to detect both *S. aureus* and *K. pneumoniae* at the same time. This method enhances the convenience of field detection for these pathogens within the milk supply chain, offering significant potential for large-scale screening and diagnosis even with limited resources.

## 2. Materials and Methods

### 2.1. Bacterial Strains

Twenty bacterial strains, including four *K. pneumoniae* strains, four *S. aureus* strains, and twelve other foodborne pathogens (Table 1), were utilized. Tryptone soy broth (TSB; Landbridge, Beijing, China) was used for the culture of *K. pneumoniae* and *V. cholerae*. Alkaline peptone water (APW; Landbridge, Beijing, China) with 3% NaCl was used for the culture of *V. parahaemolyticus* and *V. harveyi*. Luria-Bertani broth (LB; Lanqiao, Beijing, China) was used for the culture of other bacterial strains. All strains were cultivated at 37 °C for 18 h.

### 2.2. DNA Extraction

All of the genomic DNA templates were extracted by both approaches. Initially, a TIANamp Bacteria DNA Kit (Tiangen Biotech Co., Ltd., Beijing, China) was utilized to extract the genomic DNA according to the industrialist’s guideline. A NanoDrop-2000/2000c spectrophotometer (Thermo Fisher Scientific, Inc., Waltham, MA, USA) was used to determine the concentration of DNA. Furthermore, the DNA templates were extracted using the boiling method [24]. In short, 1 mL of bacterial culture was centrifuged at 13,400× *g* for 3 min. An amount of 100 μL of distilled water was used to resuspend the precipitate and boiled for 10 min. After 5 min centrifugation at 13,400× *g*, the supernatant can be taken as DNA template.

### 2.3. Design of RAA Primers

The RAA primers were developed according to the *nuc* gene of *S. aureus* and the *rcsA* gene of *K. pneumoniae* and were derived from previous studies [25,26]. Chemical modifications (6-carboxyfluorescein (FAM), digoxin (DIG)) were tagged to the 5′ ends of the forward primers of *S. aureus* and *K. pneumoniae*, respectively. Biotin was labeled on the 5′ end of the reverse primer of *S. aureus* and *K. pneumoniae* to promote the differential visualization on the dipstick (Table 2). Two amplification products were formed, each labeled with two distinguishable markers. Subsequently, these amplification products were detected utilizing a bi-lateral flow dipstick. Driven by capillary action, the amplification products of *S. aureus* labeled with FAM showed visible colored signals at the T1 line. Similarly, the amplification product of *K. pneumoniae* labeled with DIG should be detectable at the T2 line. All primers synthesized by Shanghai Sangon Biotechnology Co. (Shanghai, China) are utilized in this paper.

### 2.4. The Duplex RAA–LFD Assay

Use the RAA Nucleic Acid Amplification Kit (Qitian Genetic Biotechnology Co., Ltd., Wuxi, China) to perform the RAA reaction, adhering closely to the manufacturer’s instructions with minor modifications as necessary. In short, take 25 μL of rehydration buffer, 1 μL of 2.5 μM *S. aureus*-*rcsA*-RAA-LF3/*S. aureus*-*rcsA*-RAA-LR3 primers, 1 μL of 1.25 μM *K. pneumoniae*-*nuc*-RAA-LF2/*K. pneumoniae*-*nuc*-RAA-LR2 primers, and 16.5 μL DNase-free water and add them to the lyophilized RAA pellet to adjust the volume to 45.5 μL. Then, add 1 μL DNA of *K. pneumoniae*, 1 μL DNA of *S. aureus*, and 2.5 μL of 280 mM magnesium acetate solution. Incubate the mixture at 39 °C (Eppendorf, Hamburg, Germany) for 30 min. Finally, add 10 μL RAA product to the LFD pad, and 5 min later, the results can be observed with the naked eye. The software Image J 1.53k freeware (NIH Image, Bethesda, MD, USA) was utilized to perform the analysis of the test line intensity.

### 2.5. Optimization of the Duplex RAA–LFD Reaction Conditions

To enhance the efficiency of the RAA assay, the primer concentration, reaction temperature, and time were optimized. Based on the concentration of *S. aureus* primers at 100 nM, *K. pneumoniae* primers at varying concentrations (400, 350, 300, 250, 200, 150, and 100 nM) were added to identify the optimal ratio. Following the determination of the optimal primer concentration for the RAA reaction, the effects of different temperatures (25, 31, 33, 35, 37, 39, 41, 43, and 45 °C) on the RAA assay were studied. Subsequently, the optimal amplification time was determined at the most suitable primer concentration and temperature of the reaction for 0, 5, 10, 15, 20, 25, and 30 min.

### 2.6. Specificity of the Duplex RAA–LFD Assay

Four *K. pneumoniae strains*, four *S. aureus* strains, and twelve other foodborne pathogens were tested to assess the specificity of the duplex RAA–LFD to identify *K. pneumoniae* and *S. aureus*. The genomic DNAs extracted by the TIANamp Bacterial DNA Kit (Tiangen Biotech Co., Ltd., Beijing, China) were used as templates. Double-distilled water was used as the control.

### 2.7. Sensitivity of the Duplex RAA–LFD Assay

The detection limit of the duplex RAA–LFD was determined according to Yang et al. [28]. Firstly, the detection limit of the genomic DNA was evaluated using 6 × 10^6^~6 × 10^0^ fg/μL of *K. pneumoniae* G412 and *S. aureus* CDC AB91093 genomic DNA. Secondly, the *S. aureus* and *K. pneumoniae* cultures were diluted in a tenfold serial dilution to achieve culture concentrations ranging from 1.78 × 10^8^ to 1.78 × 10^1^ CFU/mL and 2.46 × 10^8^ to 2.46 × 10^1^ CFU/mL. Next, the DNA templates for the duplex RAA–LFD were extracted from *S. aureus* and *K. pneumoniae* cultures through the boiling method, which was performed to confirm the duplex RAA–LFD detection limitation for pure cultures.

### 2.8. Development of Selective Co-Growing Medium for S. aureus and K. pneumoniae

For the concurrent enrichment of *S. aureus* and *K. pneumoniae*, a multipathogen enrichment medium, a selective co-growing medium (SKII medium), was developed in this study. In accordance with the national standard for the *S. aureus* test method (GB4789.10-2016) [29] and the milk and dairy product hygienic microbiology test method (Part 9: *Klebsiella* test (SN/T2552.9-2010) [30]), the basic formulation of the initial SKII medium was as follows: peptone 20.0 g/L, tryptone 10.0 g/L, beef extract 5.0 g/L, sodium chloride 5.0 g/L, disodium hydrogen phosphate 8.0 g/L, potassium dihydrogen phosphate 2.0 g/L. The above components were dissolved and mixed with 1000 mL of distilled water, the pH was modified to 7.2, and the solution was autoclaved at 121 °C for 20 min. Different concentrations of sodium pyruvate (1, 2, 4 g/L), glucose (2, 4, 6 g/L), lithium chloride (0.5, 1, 2 g/L), and brilliant green (0.005, 0.01, 0.02 g/L) were added to the basic medium separately. Then, 10^3^ CFU/mL of *S. aureus* and *K. pneumoniae* were inoculated and incubated at 37 °C for 12 h. The OD_600_ was measured and the inhibition rate was calculated. The optimal concentrations of the accelerator were selected to determine the final formula of the SKII medium. The formula for calculating the inhibition rate was as follows:

(OD_600 control_ − OD_600 experimental_)/(OD_600 control_) × 100%

### 2.9. Analysis of the Growth Effect of S. aureus and K. pneumoniae in the SKII Medium

Firstly, non-target bacteria, including *L. monocytogenes*, *Salmonella*, *E. faecalis*, and *E. coli*, as well as two target bacteria, such as *S. aureus* and *K. pneumoniae*, were separately added to the SKII medium. The initial bacterial concentration was set at 10^3^ CFU/mL and cultured at 37 °C for 12 h. Subsequently, OD_600_ was measured. Then, *S. aureus* (10^3^ CFU/mL) and *K. pneumoniae* (10^3^ CFU/mL) were mixed and inoculated into the SKII medium for further cultivation at 37 °C. Cultures at 0, 4, 6, 8, 10, and 12 h were taken, respectively. *S. aureus* was counted with Baird-Parker Agar Base (BP; Landbridge, Beijing, China), and *K. pneumoniae* was counted with MacConkey Inositol Adonitol Carbenicillin Agar (MIAC; Landbridge, Beijing, China). Finally, two target bacteria and four non-target bacteria were co-cultured in the SKII medium at 37 °C for 12 h. The count of *K. pneumoniae* and *S. aureus* was carried out by selective media at 2 h intervals.

### 2.10. Simulated Sample Detection

Fresh milk was purchased from a supermarket in Shanghai to evaluate if the milk’s properties affected the RAA–LFD method. The fresh milk was confirmed to be free of *S. aureus* and *K. pneumoniae* through the biochemical identification method based on the Chinese National standard GB4789.10–2016 [29] and the Entry–Exit Inspection and Quarantine Industry Standard (SN/T 2552.9–2010) [30]. *K. pneumoniae* and *S. aureus* were incubated at 37 °C to the logarithmic phase. Then, 1 mL of pure cultures of *K. pneumoniae* and *S. aureus* with concentrations in the range of 2.46 × 10^0^~2.46 × 10^3^ CFU/mL and 1.78 × 10^0^~1.78 × 10^3^ CFU/mL, respectively, were added into a total volume of 23 mL of milk, respectively. The mixture was added into a 225 mL SKII selective co-increasing culture medium. Then, genomic DNA at different incubation periods of 0, 2, 4, and 6 h was extracted using the boiling method, and RAA amplification was performed. The amplified products were subsequently analyzed using test strips for observation.

### 2.11. PCR Assay

The PCR primers used in the analysis of actual samples are shown in Table 2. The PCR system was 25 μL, comprising Premix Taq, 12.5 μL; F/R, 1 μL each; DNA, 1 μL; and ddH_2_O, 9.5 μL. The amplification program was as follows: pre-denaturation for 5 min at 94 °C, denaturation for 30 s at 94 °C, annealing at 55 °C for 45 s, and extension for 1 min at 72 °C for 35 cycles, and then extension for 10 min at 72 °C. At the end of the reaction, the results were detected using 2% agarose gel.

### 2.12. Detection of Actual Samples

In total, 57 raw milk samples were collected from dairy farms in Shanghai. First, *K. pneumoniae* and *S. aureus* in the samples were analyzed for biochemical identification in accordance with national standards (GB4789.10-2016 and SN/T 2552.9-2010). Next, a 25 mL milk sample was added to 225 mL of SKII medium and incubated at 37 °C for 6 h. Then, 1 mL of the culture solution was used for DNA extraction via the boiling method and then used for the detection of the duplex RAA–LFD method and PCR method. Concurrently, the raw milk was evaluated for the compliance rate with the duplex RAA–LFD with PCR and national standard detection methods.

### 2.13. Statistical Analysis

Each experiment was repeated three times. The results of LFD were analyzed using Image J freeware (NIH Image, Bethesda, MD, USA). Values of the fitted curves: the results obtained from three repetitions of the experiment were analyzed using the software Image J freeware (NIH Image, Bethesda, MD, USA), and the average value was taken after determining the grayscale values. SPSS Statistics 20 program (IBM Corp., Chicago, IL, USA) was used to analyze the one-way ANOVA data. Statistical significance of the differences was assessed by using one-way ANOVA. The results were deemed to be significant with a *p*-value of less than 0.05

## 3. Results

### 3.1. Optimization of Primer Concentrations

**Figure 2:**
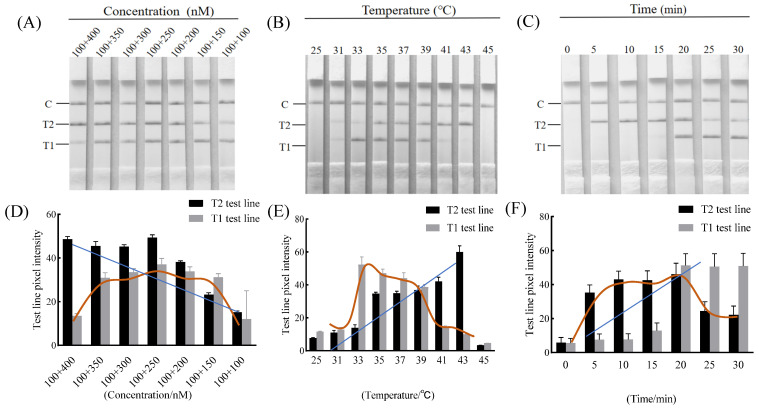
The optimization results of the duplex RAA–LFD. Primer concentration (**A**,**D**), incubation temperature (**B**,**E**), and reaction time (**C**,**F**) using genomic DNA of *S. aureus* and *K. pneumoniae.* Note: the blue and brown lines were fitted curves additionally generated using gray values.

To efficiently amplify *S. aureus* and *K. pneumoniae*, the primer concentrations of *S. aureus* and *K. pneumoniae* were optimized. As shown in Figure 2A,D, when the concentration of the *K. pneumoniae* primers decreased, the brightness of the T1 test and the T2 test system first increased and then decreased. When the concentration of *K. pneumoniae* primers was reduced to 250 nM, the signal of T1 and T2 lines was strongest. When the primer concentration was less than 250 nM, the signals for both the T1 and T2 lines decreased. Therefore, the concentration ratio of *S. aureus* and *K. pneumoniae* primers of 100:250 nM was the preferred ratio for the subsequent assays.

### 3.2. Optimization of the Duplex RAA–LFD Detection Reaction Condition

The LFD was utilized to identify the RAA products at varying temperatures. As the temperature rose, the intensity of the test line first increased and then decreased. When the temperature was 39 °C, the T1 and T2 test lines were the most suitable for brightness, with no significant difference (Figure 2B,E). Consequently, the reaction temperature of 39 °C was chosen for the following duplex RAA–LFD assay.

Then, the RAA method was executed at 39 °C for 0, 5, 10, 15, 20, 25, or 30 min. Figure 2C,F showed that the reaction time was less than 15 min, and the T1 test line was weak. As the extension of reaction time, the T1 and T2 test line signals were strongest at 20 min. After 20 min, the T2 test line signal decreased, and the brightness of the T1 line showed no significant difference (*p* > 0.05). Thus, the optimal amplification time was determined at 20 min as a result.

### 3.3. Specificity of the Duplex RAA–LFD Detection

**Figure 3:**
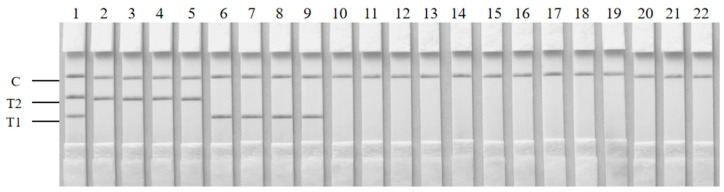
The specificity of the duplex RAA–LFD. 1—*K. pneumoniae* G412 and *S. aureus* AB91093; 2—*K. pneumoniae* G412; 3—*K. pneumoniae* G304; 4—*K. pneumoniae* G305; 5—*K. pneumoniae* G413; 6—*S. aureus* AB91093; 7—*S. aureus* G404; 8—*S. aureus* G109; 9—*S. aureus* 115; 10—*K. oxytoca* G414; 11—*S*. *saprophyticus* ATCC BAA-750; 12—*S*. *epidermidis* ATCC1228; 13—*V. cholerae* C606; 14—*L. monocytogenes* G12; 15—*L. monocytogenes* ATCC19115; 16—*P. aeruginosa* H012; 17—*E. coli* O157:H7 ATCC43889; 18—*V. parahaemolyticus* ATCC17802; 19—*V. harveyi* ATCC33842; 20—*E*. *faecalis G401*; 21—*S.* Enteritidis CMCC50041; 22—negative control.

To assess the specificity of the duplex RAA–LFD, 20 strains were investigated in this study, including four *S. aureus* strains, four *K. pneumoniae* strains, and twelve other bacterial strains (Table 1). Distilled water served as a negative control. The double-positive and single-positive results for *K. pneumoniae* and/or *S. aureus* all displayed both the appropriate test line and the control line (Figure 3). In contrast, only the control line was observed in the negative control and among non-target bacteria. This result indicated the good specificity of the duplex RAA–LFD method.

### 3.4. Sensitivity of Duplex RAA–LFD Detection

**Figure 4:**
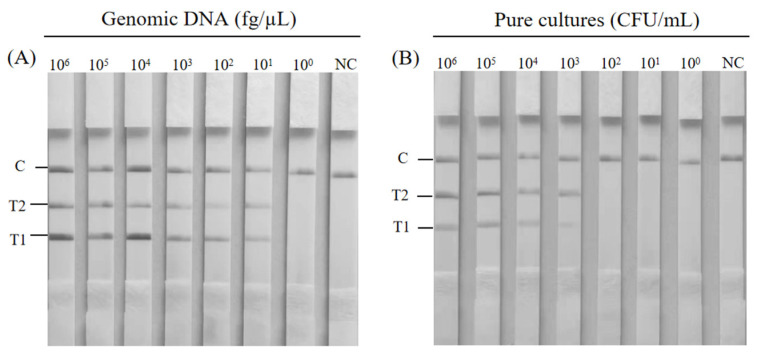
Sensitivity of the duplex RAA–LFD. (**A**) Sensitivity of the duplex RAA–LFD assay for *K. pneumoniae* and *S. aureus* genomic DNA. (**B**) Sensitivity of the duplex RAA–LFD for pure cultures of *K. pneumoniae* and *S. aureus*. NC: negative control.

The duplex RAA–LFD inspection limitation was evaluated using genomic DNA concentrations in the range of 6 × 10^6^~6 × 10^0^ fg/μL for *K. pneumoniae* and *S. aureus*. As the concentration of genomic DNA decreased, the T-line brightness of the test strip gradually became weaker (Figure 4A). Notably, at a genomic DNA concentration as low as 60 fg, although shallow, weak bands were still obtained at the T1 and T2 test lines. The results showed that the detection limit of the duplex RAA–LFD for the genomic DNA of *K. pneumoniae* and *S. aureus* was 60 fg.

Next, we established the minimum detection threshold of the duplex RAA–LFD test through the pure bacterial culture. The result is shown in Figure 4B. The color of the test lines of *K. pneumoniae* and *S. aureus* gradually faded as the concentrations decreased from 10^6^ to 10^3^ CFU/mL. The test line completely disappeared with lower concentrations (10^2^ CFU/mL and less). Therefore, the minimum detection limits of *S. aureus* and *K. pneumoniae* in pure cultures using the duplex RAA–LFD method were 1.78 × 10^3^ and 2.46 × 10^3^ CFU/mL, respectively.

### 3.5. Screening of Additives and Ratios in Co-Increasing Culture Medium

OD_600_ was measured when the culture time was 8 h. As shown in Table 3, the growth of *S. aureus* was significantly promoted with a decrease in sodium pyruvate concentration. Conversely, *K. pneumoniae* exhibited inhibited growth as the sodium pyruvate concentration increased; thus, the optimal concentration of sodium pyruvate was determined to be 1.0 g/L. The addition of glucose significantly increased the growth of both target bacteria; however, the growth-promoting effect of different concentrations of glucose on *S. aureus* was not statistically significant (*p* > 0.05). The optimal promotion effect on *K. pneumoniae* was observed at a glucose concentration of 4.0 g/L, so the concentration was determined to be 4.0 g/L. It was noted that with 0.5 g/L and 1.0 g/L lithium chloride, the growth-promoting effect on *S. aureus* and *K. pneumoniae* was not observed (*p* > 0.05). As the concentration increased, the growth of *S. aureus* was inhibited, while the growth of *K. pneumoniae* was promoted. So, the concentration of lithium chloride was determined to be 0.5 g/L. Different concentrations of brilliant green could inhibit the growth of *S. aureus* and *K. pneumoniae* obviously. Therefore, we determined the final formula of selective co-growth medium for *S. aureus* and *K. pneumoniae* (SKII medium): tryptone 10.0 g/L, peptone 20.0 g/L, beef extract 5.0 g/L, sodium chloride 5.0 g/L, disodium hydrogen phosphate 8.0 g/L, potassium dihydrogen phosphate 2.0 g/L, sodium pyruvate 1.0 g/L, lithium chloride 0.5 g/L, and glucose 4.0 g/L.

### 3.6. The Growth Effect of S. aureus and K. pneumoniae in the SKII Medium

**Figure 5:**
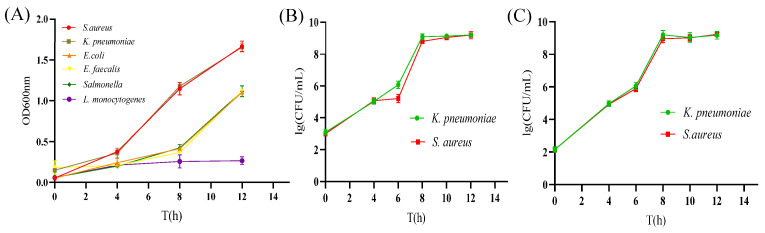
Growth effect of target bacteria under different conditions. (**A**) The growth of each bacterium in the SKII medium alone. (**B**) The growth of each bacterium in the SKII medium under the co-existence of two target bacteria. (**C**) The growth of target bacteria in the SKII medium in the presence of non-target bacteria.

The *S. aureus* and *K. pneumoniae* growth in the SKII medium was evaluated. As shown in Figure 5A, when the six strains were cultured separately in the SKII medium, the *S. aureus* and *K. pneumoniae* OD600 reached about 0.8 with incubation for 8 h. In contrast, the non-target bacteria only reached about 0.4 after 8 h culture. When *S. aureus* and *K. pneumoniae* were inoculated together into the SKII medium, it was shown that the growth of each bacterium (Figure 5B). The concentration of *S. aureus* and *K. pneumoniae* could reach 10^8^ CFU/mL after co-culture in the SKII medium for 8 h. Furthermore, when non-target bacteria and target bacteria were added to the SKII medium together, the concentration of *S. aureus* and *K. pneumoniae* could reach 10^8^ CFU/mL at 8 h (Figure 5C). The concentration of bacteria increased by about five orders of magnitude compared with the initial inoculation amount, which was comparable to the bacteria-increasing effect of target bacteria co-increasing in the absence of non-target bacteria. This result demonstrates that the SKII medium could achieve the rapid growth of both target bacteria even with the presence of non-target bacteria, which indicated that the SKII medium was suitable for the *S. aureus* and *K. pneumoniae* enrichment.

### 3.7. Assay Performance in Artificially Contaminated Milk Samples

**Figure 6:**
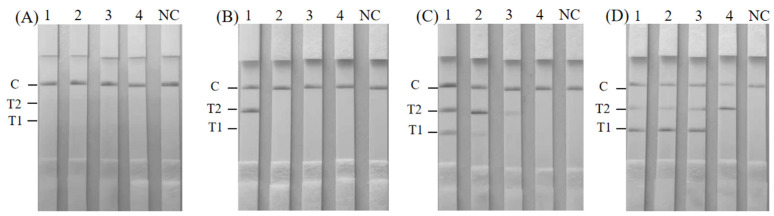
Detection of *K. pneumoniae* and *S. aureus* in simulated samples with the duplex RAA–LFD method; 1~4: 1 mL of 10^3^, 10^2^, 10^1^, and 10^0^ CFU of *K. pneumoniae* and *S. aureus* were added to 23 mL of milk and enriched for 0 h (**A**), 2 h (**B**), 4 h (**C**), and 6 h (**D**). NC: negative control.

The reliability of the duplex RAA–LFD method was verified by artificially contaminated milk samples. When *S. aureus* and *K. pneumoniae* with 10^3^, 10^2^, 10^1^, and 10^0^ CFU/mL were added to the milk sample, neither *S. aureus* nor *K. pneumoniae* was detected using the duplex RAA–LFD method in unenriched milk (Figure 6A). *S. aureus* was not detected in milk enriched for 2 h. However, *K. pneumoniae* could be detected at the initial concentration of 2.46 × 10^3^ CFU/mL (Figure 6B). Two target bacteria were successfully detected using the duplex RAA–LFD method in milk enriched for 4 h, and the detection limits were 2.46 × 10^1^ CFU/mL for *K. pneumoniae* and 1.78 × 10^2^ CFU/mL for *S. aureus* (Figure 6C). The detection limits of *S. aureus* and *K. pneumoniae* using the duplex RAA–LFD method after enrichment for 6 h were as low as 1.78 × 10^1^ and 2.46 × 10^0^ CFU/mL, respectively (Figure 6D).

### 3.8. Detection in Actual Samples

Fifty-seven raw milk samples were tested using the duplex RAA–LFD method. The performance of the duplex RAA–LFD method was evaluated by comparing its results with those obtained from traditional culture methods and PCR techniques. As shown in Table 4, *K. pneumoniae* was detected in 34 raw milk samples using the duplex RAA–LFD method, and 30 samples were positive using the conventional culture method. Four false-positive samples were detected using the duplex RAA–LFD method compared to the conventional culture method. The Total coincidence rate (TCR) for the two methods was 92.98% and the Kappa coefficient was 0.86 (*K* > 0.75), which indicated that the accuracy of the duplex RAA–LFD assay for *K. pneumoniae* was highly consistent with that of the culture method. Similarly, 32 raw milk samples were positive for *K. pneumoniae* and 25 were negative using PCR. Among the 25 negative samples using PCR, two samples were positive for *K. pneumoniae* using the duplex RAA–LFD method. Therefore, the duplex RAA–LFD method was comparable to PCR, and the TCR of the two methods was 96.49% with a Kappa coefficient of 0.93 (*K* > 0.75). Meanwhile, four raw milk samples were detected as positive for *S. aureus* using the duplex RAA–LFD method, and three were detected as positive using both the culture method (GB 4789.10-2016) and the PCR method. Compared with the culture method and PCR method, one sample was detected as a false positive using the duplex RAA–LFD method. The TCR of the duplex RAA–LFD method with the culture method and the PCR method was 98.25%, and the Kappa coefficients were both 0.85 (*K* > 0.75), indicating a high level of consistency.

## 4. Discussion

*S. aureus* and *K. pneumoniae*, as significant pathogenic bacteria, not only pose a threat to the health of dairy cows but also lead to a deterioration in milk quality, which has adverse effects on human health. Thus, there is an urgent need for the quick, on-site, and precise detection of *S. aureus* and *K. pneumoniae* to minimize the risks associated with their prevalence.

Even though nucleic acid-based assays like PCR, RT-PCR, LAMP, and RAA are the primary techniques employed for the identification of *S. aureus* and *K. pneumoniae*, these methods encounter limitations when applied in practical settings in farms and local laboratories. PCR is notably time-consuming and demands complex equipment. Nakano et al. [31] developed a LAMP assay for detecting *K. pneumoniae*, which necessitated maintaining a temperature of 68 °C for 30 min. Sheet et al. [32] introduced a LAMP method for the identification of *S. aureus*, which operated at 65 °C for 30 min and utilized four primer oligos, rendering it relatively intricate and requiring specialized temperature control equipment. Nonetheless, the duplex RAA–LFD method developed in this study requires fewer primer oligos, enabling the simultaneous detection of *S. aureus* and *K. pneumoniae* after 20 min incubation at 39 °C. Furthermore, the total detection time ranges from 45 to 85 min, encompassing genomic DNA extraction via both the boiling method (20 min) and the TIANamp Bacteria DNA Kit (60 min) for sample DNA detection. The RAA–LFD assay, in contrast to PCR and LAMP, offers a significantly quicker reaction time (saving a minimum of 1.5 h) and does not require specialized thermal cycling equipment. The real-time RPA assay for *K. pneumoniae* in urine samples, as described by Raja et al. [33], exhibited high sensitivity, detecting between 100 and 1000 copies per reaction. Geng et al. [3] established a real-time RPA method for the detection of *S. aureus* using the *nuc* gene, achieving a sensitivity of 10^2^ copies/reaction. However, grassroots laboratories often lack access to real-time monitoring instruments. The RAA–LFD assay is designed to be user-friendly for farmers or untrained personnel, making it suitable for on-the-spot detection purposes.

In this study, the duplex RAA–LFD method demonstrated excellent sensitivity, achieving a detection threshold of 60 fg of genomic DNA. The method developed in this study exhibited superior performance compared to the multiplex PCR assay established by Li et al. [34], which demonstrated a sensitivity of 1 × 10^5^ fg for *K*. *pneumoniae* and *S*. *aureus*. Furthermore, the sensitivity of the duplex RAA–LFD method for pure bacterial solutions was found to be comparable to that reported by Dung et al. [35] in the RT-PCR methods for *S. aureus* and *K*. *pneumoniae* (1.6 × 10^3^ CFU/mL), exhibiting high sensitivity. The established duplex RAA–LFD approach maintains high sensitivity levels, which helps to prevent the risk factor induced by the prevalence of *S. aureus* and *K. pneumoniae* at an early stage.

Food contaminated with pathogenic bacteria often contained only a small amount of microbes, making direct detection particularly challenging even with sensitive methods. In the practical inspection work, the enrichment culture of pathogens typically requires more than 10 to 20 h, which seriously restricts the speed of pathogen detection, and has become the technical bottleneck for rapid identification. Therefore, selective enrichment media for foodborne pathogens need to be explored, which can simultaneously increase the concentration of target pathogens in food samples to detectable levels within a short period of time through cocultivation. It is of great significance to improve the detection speed of food-borne pathogens and strengthen the prevention and control of food safety risks. Universal pre-enrichment broth, developed internationally, serves as a universal medium capable of simultaneously enriching various pathogens; however, the medium lacks selectivity and can be affected by other miscellaneous bacteria during the enrichment process [36]. In this study, a SKII selective co-growth medium was evaluated by choosing an appropriate additive concentration using a single-factor experiment, which could make the growth of *S. aureus* and *K. pneumoniae* with an initial inoculation amount of 10^3^ CFU/mL reach about 10^8^ CFU/mL after 8 h. Furthermore, in the presence of non-target bacteria, the growth of target bacteria remained unaffected, thereby fulfilling the requirements of subsequent detection.

For the specificity part, a total of 20 strains were investigated in this paper, and the results showed good specificity. Li et al. [34] established a multiplex PCR method for the simultaneous detection of *E. coli*, *Salmonella*, *K. pneumoniae*, and *S. aureus*, and a total of 15 strains were selected for the specificity test, which showed good results. Wang et al. [37] developed a duplex biosensor based on RPA and a three-segment lateral flow strip (LFS) for *V. cholerae* and *V. vulnificus*; 19 strains were selected for specificity testing and also showed good specificity. Samples of artificially contaminated milk were used to validate the accuracy of the duplex RAA–LFD method. The results indicated that 1.78 × 10^1^ CFU/mL for *S. aureus* and 2.46 CFU/mL for *K. pneumoniae* could be detected using the duplex RAA–LFD method after 6 h of enrichment. This limit of detection was 10 times higher than that of 9 × 10^2^ CFU/mL for detecting *S. aureus* in artificially contaminated food samples via LAMP [32]. The inspection limitation of duplex RAA–LFD for *K. pneumoniae* was comparable to that of Feng et al. [38], which demonstrated the same limit of detection. Therefore, the duplex RAA–LFD technique developed in our research exhibits good specificity, sensitivity, and rapid detection capabilities, which is suitable for broad applications.

In the detection of 57 raw milk samples, four and one false-positive results for *K. pneumoniae* and *S. aureus* were detected using the duplex RAA–LFD approach compared with the culture method. The TCR of the duplex RAA–LFD method and culture method for *K. pneumoniae* and *S. aureus* were 92.98% and 98.25%, respectively. The incorrect picking of colonies, the presence of non-culturable cells, and the existence of dead cells may be the reasons why more positive samples were detected using the duplex RAA–LFD method than the culture method [28]. Similarly, the duplex RAA–LFD method yielded two and one false-positive results for *K. pneumoniae* and *S. aureus*, respectively, when compared to the PCR method. Consequently, the TCR of the duplex RAA–LFD and PCR methods for *K. pneumoniae* and *S. aureus* were 96.49% and 98.25%, respectively. The increased incidence of false-positive results observed with the duplex RAA–LFD method may be due to the fact that the RAA method is more affected by the food matrix compared to the PCR method [39]. Moreover, the results obtained from the duplex RAA–LFD method and the PCR method were different but the compliance rate of the duplex RAA–LFD method and the PCR method for the detection of *K. pneumoniae* and *S. aureus* still reached more than 96.00%, with high consistency (K > 0.75). This indicated that the RAA–LFD method had a high detection accuracy. However, the duplex RAA–LFD method eliminates the need for laborious separation, purification, and biochemical identification steps. It also does not rely on costly and cumbersome instrumentation, and the entire detection process is simple and rapid, which is conducive to the detection of *K. pneumoniae* and *S. aureus* in field environments with limited resources and equipment.

## 5. Conclusions

Based on the *rcsA* and *nuc* genes, a duplex RAA–LFD method was first established for the simultaneous detection of *S. aureus* and *K. pneumoniae* in milk. The duplex RAA–LFD assay is characterized by being highly sensitive and specific, requiring neither specialized equipment nor complex procedures. The detection results of raw milk showed that the duplex RAA–LFD method had high consistency with the microbial biochemical identification method and PCR method. The duplex RAA–LFD test is suitable for on-the-spot detection of *S. aureus* and *K. pneumoniae*. Moreover, a SKII medium was developed that can enrich both *S. aureus* and *K. pneumoniae* in a short period of time for rapid bacterial growth, which is important for enhancing the prevention and control of food safety risks.

## Figures and Tables

**Table 1:** Bacterial strains.

Species	Strain	Source
*Klebsiella pneumoniae*	G412	Isolated from dairy farms
*Klebsiella pneumoniae*	G304	Isolated from dairy farms
*Klebsiella pneumoniae*	G305	Isolated from dairy farms
*Klebsiella pneumoniae*	G413	Isolated from dairy farms
*Staphylococcus aureus*	AB91093	CDC
*Staphylococcus aureus*	G404	Isolated from dairy farms
*Staphylococcus aureus*	G109	Isolated from dairy farms
*Staphylococcus aureus*	G115	Isolated from dairy farms
*Klebsiella oxytoca*	G414	Isolated from dairy farms
*Staphylococcus saprophyticus*	ATCC BAA-750	ATCC
*Staphylococcus epidermidis*	ATCC1228	ATCC
*Vibrio cholerae*	C6067	Preserved in our laboratory
*Listeria monocytogenes*	G12	Isolated from dairy farms
*Listeria monocytogenes*	ATCC19115	ATCC
*Pseudomonas aeruginosa*	H012	Preserved in our laboratory
*Escherichia coli O157:H7*	ATCC43889	ATCC
*Vibrio parahaemolyticus*	ATCC17802	ATCC
*Vibrio harveyi*	ATCC33842	ATCC
*Enterococcus faecalis*	G401	Isolated from dairy farms
*Salmonella* Enteritidis	CMCC50041	CMCC

ATCC, American Type Culture Collection; CMCC, China Center for Medical Culture Collection; CDC, Centers for Disease Control and Prevention, Shanghai, China.

**Table 2:** The primers used in this study.

Method	Pathogen	Primers	Sequence (5′–3′)	Product Size (bp)	Cite
RAA–LFD	*Klebsiella pneumoniae*	*rcsA*-LF3	DIG-TGTATTTTCTTTTTAATATTGCCTTTATGCG	137	[25]
		*rcsA*-LR3	Biotin-TTGTCACTGAGTAAAACAGAATCAAATATGC		
	*Staphylococcus aureus*	*nuc*-LF2	6-FAM-GACAAAGGTCAAAGAACTGATAAATATGGA	159	[26]
		*nuc*-LR2	Biotin-TTCACTTTTTCTTAAAAGTTGTTCATGTGT		
PCR	*Klebsiella pneumoniae*	*khe*-F	TGATTGCATTCGCCACTGG	428	[26]
		*khe*-R	GGTCAACCCAACGATCCTG		
	*Staphylococcus aureus*	*nuc*-F	CTGGCATATGTATGGCAATTGTT	664	[27]
		*nuc*-R	TATTGACCTGAATCAGCGTTGTCT		

**Table 3:** The growth influence of ingredients added in culture on target bacteria.

Additive	Dose (g/L)	Inhibition Rate (%)	
		*S. aureus*	*K. pneumoniae*
Sodium pyruvate	1.0	−8.45 ± 0.011 ^a^	−39.30 ± 0.027 ^a^
	2.0	−2.12 ± 0.006 ^b^	−37.46 ± 0.045 ^a^
	4.0	−3.54 ± 0.005 ^b^	15.33 ± 0.007 ^b^
Glucose	2.0	−20.81 ± 0.195 ^a^	−31.22 ± 0.009 ^a^
	4.0	−16.24 ± 0.035 ^a^	−37.92 ± 0.024 ^ab^
	6.0	−21.41 ± 0.101 ^a^	−34.35 ± 0.004 ^b^
Lithium chloride	0.5	−1.17 ± 0.006 ^a^	−11.45 ± 0.046 ^ab^
	1.0	−1.49 ± 0.005 ^a^	−8.30 ± 0.029 ^a^
	2.0	5.73 ± 0.006 ^b^	−21.20 ± 0.074 ^b^
Brilliant green	0.005	58.10 ± 0.097 ^a^	35.86 ± 0.043 ^a^
	0.01	38.78 ± 0.009 ^b^	15.33 ± 0.007 ^b^
	0.02	21.13 ± 0.030 ^b^	16.73 ± 0.024 ^c^

Different letters represent that the same column and the same additive in the growth difference are statistically significant, *p* < 0.05; “±” represents the margin of error.

**Table 4:** Detection of practical samples using the duplex RAA–LFD assay and PCR compared with the biochemical identification method.

Strains	*Klebsiella pneumoniae*		*Staphylococcus aureus*	
	Duplex RAA–LFD /SN/T 2552.9–2010	Duplex RAA–LFD/PCR	Duplex RAA–LFD /GB 4789.10–2016	Duplex RAA–LFD /PCR
Raw milk (57)	34/30	34/32	4/3	4/3
TP	30	32	3	3
TN	23	23	53	53
FP	4	2	1	1
FN	0	0	0	0
PPV (%)	88.24	94.12	75.00	75.00
NPV (%)	100.0	100.0	100.0	100.0
Sensitivity (%)	100.0	100.0	100.0	100.0
Specificity (%)	85.19	92.00	98.15	98.15
TCR (%)	92.98	96.49	98.25	98.25
K	0.86	0.93	0.85	0.85

Note: TP: True positive; TN: True negative; FP: False positive; FN: False negative; PPV (Positive predictive value) = TP/(TP + FP) × 100%; NPV (Negative predictive value) = TN/(TN + FN) × 100%; Sensitivity = TP/(TP + FN) × 100%; Specificity = TN/(TN + FP) × 100%; TCR (Total coincidence rate) = (TP + TN)/total sample quantity × 100%; K (Kappa coefficient): K ≥ 0.75 (high consistency).

## Data Availability

The original contributions presented in the study are included in the article, and further inquiries can be directed to the corresponding authors.
